# Antimicrobial therapy of community-acquired pneumonia during stewardship efforts and a coronavirus pandemic: an observational study

**DOI:** 10.1186/s12890-022-02178-6

**Published:** 2022-10-14

**Authors:** Bjørn Waagsbø, Morten Tranung, Jan Kristian Damås, Lars Heggelund

**Affiliations:** 1 St. Olavs Hospital, Regional centre for disease control in Central Norway Regional Health Authority, Trondheim University hospital, Trondheim, Norway; 2 Department of Clinical and Molecular Medicine, Norwegian University of Science and Technology, Trondheim, Norway; 3 Central Norway Hospital Pharmacy Trust, Trondheim, Norway; 4 Department of Infectious Diseases, St. Olavs Hospital, Trondheim University hospital, Trondheim, Norway; 5 Centre of Molecular Inflammation Research, department of Clinical and Molecular Medicine, NTNU, Trondheim, Norway; 6 Department of Internal Medicine, Drammen Hospital, Vestre Viken Hospital Trust, Drammen, Norway; 7 Department of Clinical Science, Faculty of Medicine, University of Bergen, Bergen, Norway

**Keywords:** Pneumonia, Community-acquired pneumonia, Aetiology, Microbiology, Antimicrobial stewardship, Antimicrobial therapy

## Abstract

**Background:**

Community-acquired pneumonia (CAP) is the most frequent infection diagnosis in hospitals. Antimicrobial therapy for CAP is depicted in clinical practice guidelines, but adherence data and effect of antibiotic stewardship measures are lacking.

**Methods:**

A dedicated antibiotic team pointed out CAP as a potential target for antimicrobial stewardship (AMS) measures at a 1.000-bed, tertiary care, teaching university hospital in Norway from March until May for the years 2016 throughout 2021. The aim of the AMS program was to increase diagnostic and antimicrobial therapy adherence to national clinical practice guideline recommendations through multiple and continuous AMS efforts. Descriptive statistics were retrospectively used to delineate antimicrobial therapy for CAP. The primary outcomes were proportions that received narrow-spectrum beta-lactams, and broad-spectrum antimicrobial therapy.

**Results:**

1.112 CAP episodes were identified. The annual proportion that received narrow-spectrum beta-lactams increased from 56.1 to 74.4% (p = 0.045). Correspondingly, the annual proportion that received broad-spectrum antimicrobial therapy decreased from 34.1 to 17.1% (p = 0.002). Trends were affected by the coronavirus pandemic. Mortality and 30-day readmission rates remained unchanged. De-escalation strategies were frequently unutilized, and overall therapy duration exceeded clinical practice guideline recommendations substantially. Microbiologically confirmed CAP episodes increased from 33.7 to 56.2% during the study period.

**Conclusion:**

CAP is a suitable model condition that is sensitive to AMS measures. A continuous focus on improved microbiological diagnostics and antimicrobial therapy initiation is efficient in increasing adherence to guideline recommendations. There is an unmet need for better antimicrobial de-escalation strategies.

**Supplementary Information:**

The online version contains supplementary material available at 10.1186/s12890-022-02178-6.

## Introduction

Community-acquired pneumonia (CAP) is a frequent infection of the lower respiratory tract, each year accounting for millions of hospitalizations and significant morbidity and mortality worldwide [1]. A majority of cases is caused by well-known respiratory tract pathogens, but microbiological confirmation is somewhat hampered by the lack of sample harvesting, and the sensitivity and specificity of respiratory tract secretions [2].

Antimicrobial therapy is considered essential for the proper management of CAP [3]. Due to high numbers of cases, frequent misdiagnosed aetiology, and the associated mortality risk, CAP has the potential to propel antimicrobial consumption. For this reason, diagnostic and antimicrobial stewardship measures are justified, aiming at the recognition of exposure settings, acquisition, risk factors, severity assessment, and the timely initiation and de-escalation of antimicrobial therapy [4].

Antimicrobial resistance (AMR) among several respiratory tract pathogens to commonly used antimicrobials are increasing worldwide [5]. However, in a few countries, AMR still remains at relatively modest levels, as reported from particularly Scandinavian countries [6]. This favorable situation is on the other hand vulnerable to consequences of unjustified broad-spectrum antimicrobial prescriptions.

A national clinical practice guideline for the management of CAP was established in Norway in 2013. Therapy recommendations were largely consistent with those from other European countries, targeting frequently isolated respiratory pathogens. For non-severe CAP, benzyl penicillin was recommended, and for severe CAP, the addition of gentamicin or alternatively cefotaxime in monotherapy. A revision in 2020 left most recommendations unchanged, but added that CAP managed in intensive care settings should receive cefotaxime in combination with ciprofloxacin [7].

The objective of this study was to investigate antimicrobial therapy for CAP in a low AMR setting at a regional, university teaching hospital over six consecutive years during multiple and continuous antimicrobial stewardship efforts, and the interruption of a coronavirus pandemic.

## Patients and methods

### Study setting

A 1.000-bed university teaching hospital in Norway, accepting all patients, included patients with covid-19.

### Study population

For each year from 2016 to 2021 we used hospital administration data to retrospectively identify patients ascribed with a pneumonia diagnosis at discharge. Criteria for identification were ICD-10 J13 to J18.9 as a primary diagnosis, months from March throughout May, age above 18 years, and admission to hospital antimicrobial therapy at the medical or pulmonary ward, or the intensive care unit. In order to minimize selection bias, cases ascribed with a viral aetiology to lower respiratory tract infection were not included in the criteria for identification. We chose months from March throughout May to reduce influenza virus disease influence, and to address regular hospital staffing outside of summer holiday circumstances.

To include definite CAP cases and an unequivocal study cohort, several exclusion criteria were established. Among these were hospital-acquired or ventilator-associated infections, aspiration pneumonias, pneumonia in nursing home residents, in the returned traveler, in the immunocompromised patient, and in patients diagnosed with chronic obstructive pulmonary disease, or infection in the lower respiratory tract other than pneumonia. We also excluded cases if length of stay exceeded 28 days. Criteria for clinically significant immunosuppression that were used in a recent randomized clinical trial on CAP management, were applied [8]. Exclusion criteria secured that cases investigated were likely to be diagnosed and treated according to the CAP clinical practice guideline recommendations.

### Study outcomes

The primary outcome of the study was proportions of CAP episodes that received antimicrobial therapy in accordance with clinical practice guideline recommendations. This included proportions each year that received narrow-spectrum beta-lactams, and proportions that received broad-spectrum antimicrobial therapy. The clinical practice guideline recommendations are provided in the appendix.

### Data collection

A study group, consisting of clinical pharmacists and an infectious diseases physician performed data collection and evaluation after each study year. Both discharge letters, medical records, radiological evaluations, and laboratory data were recovered and analyzed.

We have previously published data on an antimicrobial diagnostic strategy in the emergency room (ER) setting targeting respiratory tract specimen sampling [9]. This intervention included CAP episodes from 2016 to 2018. We then wanted to expand the knowledge on CAP management in our institution, in order to evaluate the impact of stewardship efforts on antimicrobial therapy for the ensuing years.

### Laboratory procedures

In the laboratory, we used conventional techniques to secure microbiological confirmations from cultures. We also applied nucleic acid amplification techniques (NAAT) whenever this was required from the attending physician or laboratory personnel according to laboratory practice. All lower respiratory tract specimens were subjected to microscopy by a microbiologist before cultivation. The polymorphonuclear to squamous epithelial cell ratios were used to determine indications for cultivation. Laboratory personnel was uninformed of the study, seeking to maintain everyday clinical laboratory service.

### Stewardship measures

Antimicrobial stewardship measures aiming at several therapy features were disseminated throughout the institution during the study period. These included, but were not limited to, both diagnostic and therapeutic aspects of CAP management. Of particular focus were disease severity assessment, timely administration of antimicrobial therapy, selection of empirical regimens, de-escalation strategies, targeted antimicrobial therapy, oral regimen conversion, and the overall therapy duration. Most efforts were directed at increasing adherence to national clinical practice guideline recommendations. A revised and updated national clinical practice guideline was available from 2020 [7], and relevant contents were transferred to local clinical practice management procedure that same year.

All measures and areas of focus were applied to clinicians through a dedicated, institutional antibiotic team consisting of an infectious disease physician, clinical microbiologist, and clinical pharmacists. Initially we offered tutoring sessions to on-call physicians in selected departments, targeting CAP management recommendations. The next year and thereafter we conducted monthly visits to the ER and selected departments, addressing on-call physicians, ward-level physicians, and nurse staffing with CAP management recommendations. We also provided yearly statistics on performance for all selected departments, and AMR reports that compared narrow-spectrum to broad-spectrum regimens. Interventions are described in detail in the appendix.

### Antimicrobial therapy

We defined penicillinase-susceptible penicillins, amoxicillin, and doxycycline narrow-spectrum antimicrobials, whilst beta-lactams co-formulated with enzyme inhibitors, cephalosporins, carbapenems, and quinolones, broad-spectrum antimicrobials. The addition of gentamicin has been used for decades for severe CAP in Nordic countries, especially in cases of structural pulmonary disease. Macrolides has traditionally been indicated to cover for atypical aetiology or in case of beta-lactam allergy for non-severe CAP. According to clinical practice guideline recommendations, broad-spectrum antimicrobial therapy for CAP was indicated in cases of known renal failure, sepsis syndrome with organ dysfunction at admission, or if suspicion of CAP caused by an extended spectrum beta-lactamase (ESBL) producing pathogen. In the absence of these conditions, we defined administered broad-spectrum antimicrobial therapy unjustified.

Therapy days were measured as complete days if ≥ 50% of the dosages were administered for that 24-hour dose interval. Duration of therapy was assessed using number of days prescribed with empirical and targeted antimicrobial therapy from the first antimicrobial therapy day after hospital admittance to the registered last day of therapy after hospital discharge.

### Statistical analyses

Descriptive statistics were used to delineate antimicrobial therapy instituted for CAP. Empirical and targeted antimicrobial therapy were compared between study years. Especially, adherence to national clinical practice guidelines was assessed for each year. A chi-square association model was used to detect change in therapy regimens and aetiology between study years. For the comparison of means between study years, a one-way ANOVA was employed. P < 0.05 was used as clinical significance level.

### Ethical considerations

The study was approved by the Regional Committee for Medical and Health Research Ethics (REK 2017/1439), data protection officials, and the hospital administration. Inclusion of patients was performed without consent, on the basis of a retrospective study design, assuming that all included cases received best practice as described in national clinical practice guideline recommendations. Antimicrobial stewardship measures were aimed to optimize adherence to clinical practice guidelines.

## Results

### Patient characteristics

Over the six-year study period, 1.839 unique hospital discharges were identified meeting the identification criterion of pneumonia as a primary diagnosis. Of these, 726 fulfilled one or more exclusion criteria and were rejected from further analyses. The remaining portion constituted 1.112 unique episodes of adult, community-acquired, hospitalized pneumonia, devoid of clinically relevant immunocompromise or chronic obstructive pulmonary disease, and of which 91% were radiologically confirmed. Clinical signs and symptoms, microbiological confirmations, and the clinical response to the instituted antimicrobial therapy were used to verify CAP-diagnosis in radiologically unconfirmed cases. Descriptive statistics for the patient population are presented in Table 1. Included episodes did not differ between years for all comparisons.

### Antimicrobial therapy

About 20% of CAP episodes had received antimicrobial therapy before hospital admission, with variations between years ranging from 17 to 23%. Prehospital prescriptions were mainly narrow-spectrum beta-lactams such as phenoxymethylpenicillin, amoxicillin, and dicloxacillin adding up to 54%, trimethoprim-sulfametoxazole to 14%, and ciprofloxacin to 12%.

**Fig. 1:**
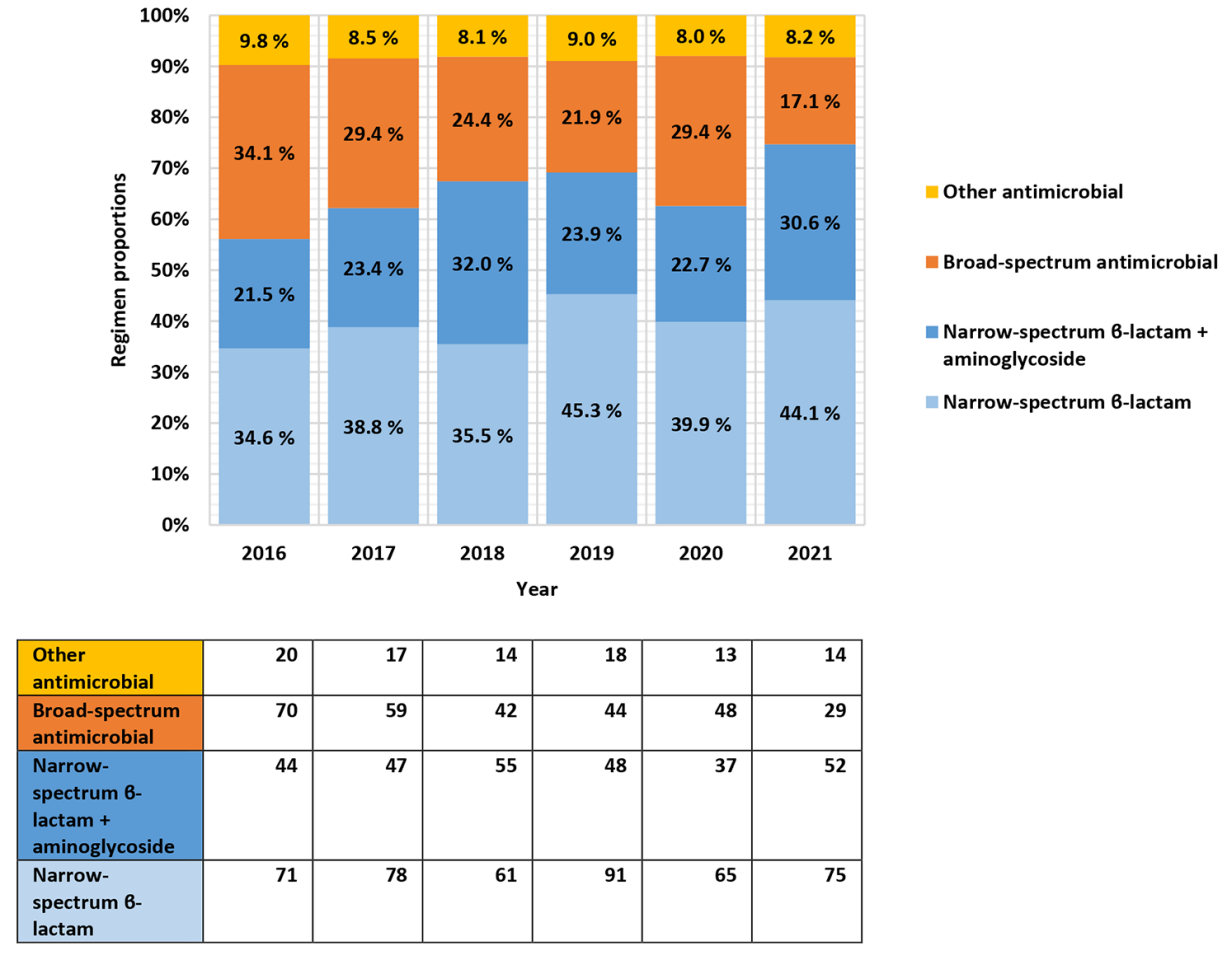
Proportions of empirical regimens for CAP per year

Empirical antimicrobial therapy were initiated to all admitted cases on suspicion of bacterial CAP. Interestingly, a total of 16 different generic antibiotic substances were administered as the initial drug of choice, of which the majority were narrow-spectrum beta-lactams. The empirical antimicrobial therapy used for each study year is presented as proportions in Fig. 1.

As described in the method section an antimicrobial stewardship team launched several measures to actively improve diagnostic and therapy strategies. Using an association model for all regimens, a statistically significant increase in proportions that received narrow-spectrum beta-lactams, including beta-lactams combined with an aminoglycoside (Pearson chi-square χ^2^ = 17.3; df 5; p = 0.004) was observed throughout the study period. The proportion increased from 56.1 to 74.4%, which represented a relative change of 32.6%. Narrow-spectrum beta-lactams were frequently combined with gentamicin, at proportions ranging from 21.5 to 32.0% between years, and for a mean duration of 2.8 days (95% CI 2.2–3.4) for all years. The proportions that annually received gentamicin did not significantly differ (Pearson chi-square χ^2^ = 9.3; df 5; p = 0.097).

Correspondingly, proportions that received broad-spectrum cefotaxime, ceftriaxone, piperacillin-tazobactam or any carbapenem declined significantly from 34.1 to 17.1% (Pearson chi-square χ^2^ = 19.4; df 5; p = 0.002), which constitute a relative reduction of 49.9%. For all years combined, broad-spectrum antimicrobial therapy was administered in cases of established renal disease, sepsis-syndrome, management in the ICU setting, beta-lactam allergy, and ESBL-producing pathogen, in 12.2%, 11.4%, 6.1%, 6.0% and 0.2%, respectively. Unjustified broad-spectrum antimicrobial therapy constituted 64.1% of episodes for all years. However, a statistically significant decline per year was observed (Pearson chi-square χ^2^ = 16.8; df 5; p = 0.005). There was a notable discrepancy in empirical antimicrobial therapy for the year 2020, as cefotaxime were frequently administered. Mortality and readmission rates were comparable between years, as described in Table 1.

**Table 1:** Patient characteristics and outcomes for included CAP episodes

n	Patients	205	201	172	201	163	170	1112	0.086
Age	Mean (years)	70,5	68,5	72,8	69,0	71,1	70,4	70,3	0.196
Age group	< 50 years	11,7%	14,4%	6,4%	14,4%	11,7%	10,6%	11,7%	
	50–75 years	38,0%	42,3%	40,7%	38,8%	35,0%	38,8%	39,0%	
	> 75 years	50,2%	43,3%	52,9%	46,8%	53,4%	50,6%	49,3%	0.966
Gender	Male (%)	43,9%	51,7%	48,8%	44,8%	41,7%	41,8%	45,5%	0.299
	Female (%)	56,1%	48,3%	51,2%	55,2%	58,3%	58,2%	54,5%	
Comorbidities*	Number of conditions (median)	3	3	3	3	3	3	3	0.914
	Charlson comobidity index (median)	4	4	4	4	4	4	4	
CRB65	0–1 (%)	141 (69%)	133 (66%)	128 (74%)	147 (73%)	119 (73%)	115 (68%)	783 (70%)	
	2 (%)	51 (25%)	49 (24%)	38 (22%)	46 (21%)	35 (21%)	49 (29%)	268 (24%)	
	3–4 (%)	13 (6%)	19 (9%)	6 (3%)	8 (4%)	9 (6%)	6 (4%)	61 (5%)	0.570
ICU	Admittance (n, %)	11 (5%)	18 (9%)	14 (8%)	7 (3%)	9 (6%)	9 (5%)	68 (6%)	0.222
Ventilation	Invasive (n, %)	7 (3%)	8 (4%)	6 (3%)	9 (4%)	6 (4%)	6 (4%)	42 (4%)	0.995
	NIPPV (n, %)	18 (9%)	16 (8%)	17 (10%)	22 (11%)	16 (10%)	20 (12%)	109 (10%)	0.304
Sepsis	Without shock (n, %)	22 (11%)	16 (8%)	23 (13%)	16 (8%)	16 (10%)	14 (8%)	107 (10%)	0.740
	Septic shock (n, %)	2 (1%)	3 (1%)	3 (2%)	5 (2%)	4 (2%)	5 (3%)	22 (2%)	0.757
Length of stay	Mean (days), and (95% CI)	7.4 (6.6–8.2)	7.3 (6.7–7.8)	7.0 (6.3–7.7)	6.7 (6.2–7.3)	7.8 (7.1–8.4)	7.2 (6.5–7.8)	7.2 (6.9–7.5)	0.342
Re-admission	30 day (n, %)	16 (7.8)	11 (5.5)	6 (3.5)	12 (5.9)	6 (3.7)	8 (4.7)	59 (5.3)	0.860
Mortality	In-hospital (n, %)	22 (11%)	21 (10%)	16 (9%)	20 (10%)	18 (11%)	20 (12%)	117 (11%)	0.984
	30 day (n, %)	28 (14%)	26 (13%)	24 (14%)	22 (11%)	29 (18%)	23 (14%)	152 (14%)	0.512
	90 day (n, %)	56 (27%)	46 (23%)	42 (24%)	40 (20%)	39 (24%)	44 (26%)	267 (24%)	0.498

### Aetiology and resistance

**Fig. 2:**
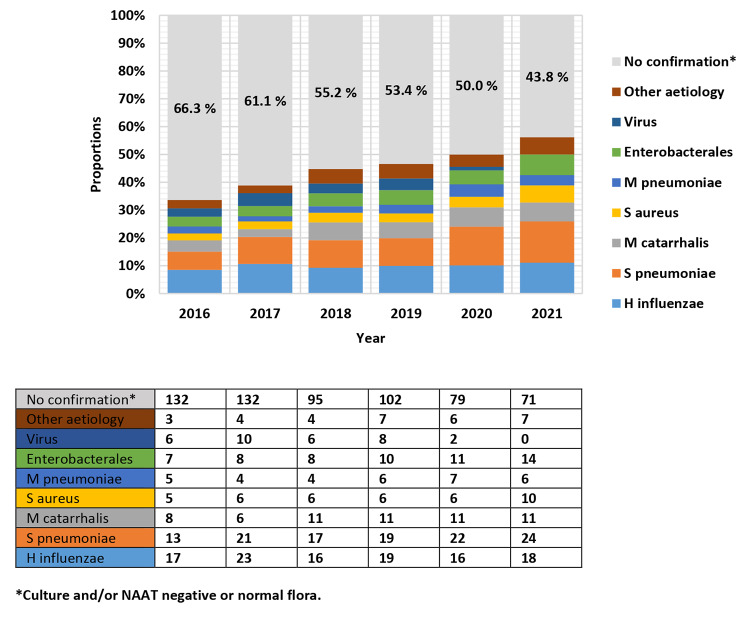
Definite CAP aetiology per year

Proportions of CAP episodes that were microbiologically confirmed increased from 33.7 to 56.2% during the study period. A Pearson Chi-square correlation model returned a statistically significant increase between years (χ2: 23.431, df5, p < 0.001). Likewise, proportions of CAP episodes devoid of microbiological confirmation decreased accordingly. Respiratory tract pathogens that were most frequently detected were *S. pneumoniae*, *H. influenza*, and *M. catarrhalis* that accounted for 23.8%, 22.4% and 11.9% for all years, respectively. The use of nuclear acid amplification (NAAT) tests identified 7–17% of CAP aetiology depending on inclusion year. CAP episodes with a clinically relevant positive blood culture also remained unchanged, as range spanned from 3.2 to 4.8% between years. Aetiology for CAP is presented in Fig. 2.

Proportions of microbiologically confirmed cases with an AMR phenotype were assessed. All the 116 of 116 (100.0%) *S. pneumoniae* isolates remained fully susceptible to penicillin. Among *H. influenzae* cases 19 of 109 (17.4%) were resistant to ampicillin. No cases of methicillin resistant *S. aureus* (MRSA) were reported, and among *Enterobacterales*, 2 of 53 cases (3.8%) were ESBL positive. No cases of multiresistant *P. aeruginosa* or *Legionella pneumophila* were reported.

### CAP severity

**Fig. 3:**
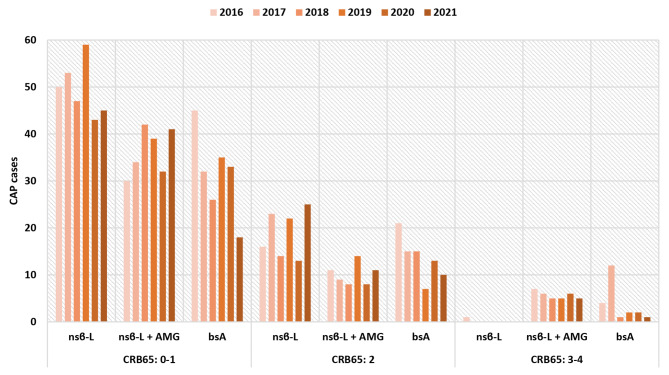
Empirical antimicrobial therapy stratified by CRB65-category. Abbrevations: nsβ-L = narrow-spectrum betalactam, AMG = aminoglycoside, bsA = broad-spectrum antimicrobial

Disease severity at presentation was assessed for all CAP episodes. We retrospectively applied CRB65-criteria and stratified this to the various empirical antimicrobial therapy regimens. CAP episodes that scored 0–1, 2 or 3–4 constituted 70.4%, 24.1% and 5.5%, respectively, with minimal variation between years. In the CRB65 0–1 category, a substantial proportion of CAP episodes received a broad-spectrum antimicrobial. However, this proportion declined in the study period, although statistically insignificant. Among episodes presenting within the CRB65 3–4 category, very few received narrow-spectrum beta-lactam therapy only, while the majority received narrow-spectrum beta-lactam in combination with an aminoglycoside. Figure 3 presents the administered empirical antimicrobial regimens.

### Antimicrobial de-escalation

De-escalation strategies were assessed for CAP episodes that were microbiologically confirmed and received broad-spectrum empirical antimicrobial therapy. The assessment included the timely transition from empirical to targeted antimicrobial therapy, and the transition from intravenous to oral administration route. For all CRB65 groups, broad-spectrum antimicrobial therapy was continued in 66.4% of episodes, without the transition to a more preferred narrow-spectrum therapy, although this would have been feasible in regard to antimicrobial susceptibility (AMS) testing. In 24.6% of episodes, de-escalation was applied, however, on average 1.9 days beyond the AMS reporting time point. For most CAP episodes an intravenous to oral conversion was feasible, but not performed at the AMS reporting time point. Table 2 summarizes the strategies for antimicrobial therapy from the time point of AMS testing being reported.

**Table 2:** Antimicrobial strategies used for microbiologically confirmed CAP episodes that received broad-spectrum antimicrobial therapy

	CRB65 0–1	CRB65 2	CRB65 3–4	All groups
**n**	**88**	**35**	**11**	**134**
Strategy instituted at point of AMS reporting				
Broad-spectrum to narrow-spectrum B-lactam transition	28,4%	20,0%	9,1%	24,6%
Transition not recommended	6,8%	8,6%	27,3%	9,0%
Transition feasible, but continued broad-spectrum antimicrobial	64,8%	71,4%	63,6%	66,4%
Administration form instituted at point of AMS reporting				
Intravenous to oral conversion	20,5%	14,3%	0,0%	17,2%
Conversion not possible	11,4%	17,1%	45,5%	15,7%
Conversion possible, but not performed	68,2%	68,6%	54,5%	67,2%

### Duration of therapy

**Fig. 4:**
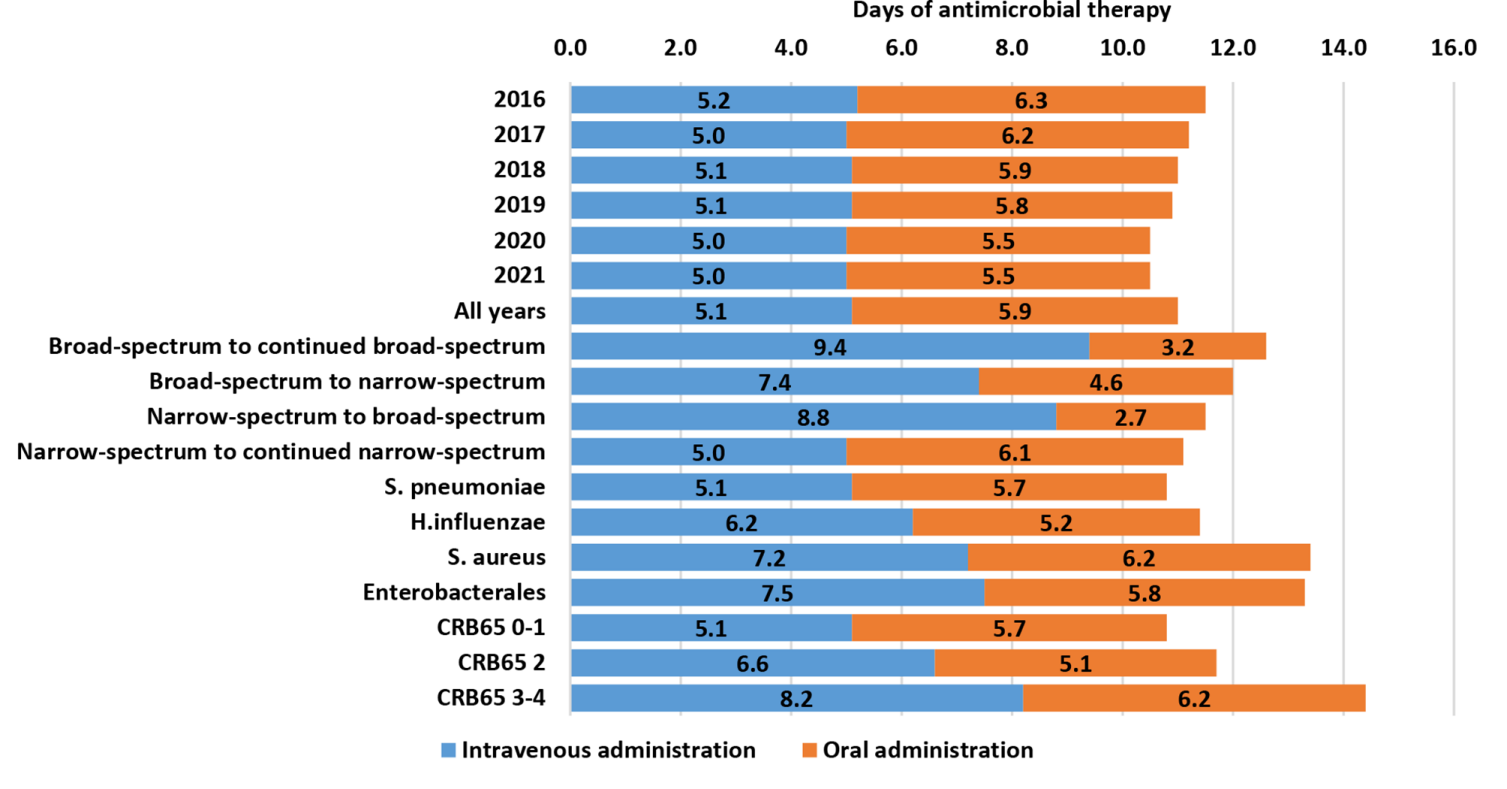
Antimicrobial therapy duration stratified by years, antimicrobial therapy, aetiology and disease severity

Prehospital therapy was excluded from duration analyses because of consistently unreliable information. Figure 4 describes the therapy duration for all included CAP episodes. The combined mean duration of therapy was 11.0 days (95% CI 10.9–11.1). Using a one-way ANOVA comparison of means returned a small but statistically significant reduction in total antimicrobial therapy duration from 11.5 to 10.2 days (p < 0.001). This reduction was largely attributed to shorter oral regimens.

## Discussion

In this retrospective observational study of adult, hospitalized CAP in immunocompetent, non-COPD patients, we have investigated trends in antimicrobial therapy for six consecutive years during antimicrobial and diagnostic stewardship measures. A significant increase in proportions that received narrow-spectrum beta-lactams from 56 to 75%, and correspondingly, a decrease in proportions that received broad-spectrum antimicrobial therapy from 34 to 17% were observed. During the coronavirus pandemic year 2020, a larger extent of bacterial CAP episodes initially received third generation cephalosporins. Overall, de-escalation strategies were frequently unutilized, and therapy duration exceeded recommendations substantially. Increased focus on routine microbiological diagnostic strategies increased the absolute microbiological yield from 33.7 to 56.2% without the implementation of new laboratory methods. We found low and comparable rates of AMR. Mortality and re-admission rates between years remained unchanged, underscoring that stewardship measures seem justified in order to increase adherence to therapy guideline recommendations.

Narrow-spectrum regimens are recommended as first line therapy for hospitalized, non-severe CAP in several clinical practice guidelines [3, 7, 10, 11]. These include penicillinase-susceptible penicillins, amoxicillin, or doxycycline, whilst beta-lactams co-formulated with enzyme inhibitors, cephalosporins, carbapenems, quinolones and macrolides, are generally considered broad-spectrum antimicrobials. We have here reported a statistically significant increased use of narrow-spectrum, warranted beta-lactams. We believe this increase is due to the continued efforts from stewardship measures to influence on diagnostic and therapy management to this strategic patient group, as shown previously by others [8, 12]. Of note, the increase in narrow-spectrum antimicrobial therapy did not result in altered mortality or re-admission rates.

CAP management is an important subject for antimicrobial stewardship [4]. In a recent, cluster randomized intervention study from twelve hospitals in the Netherlands, broad-spectrum antimicrobial therapy for non-severe CAP were decreased from 6.5 to 4.8 days, which represents a relative reduction of 27% [8]. The 90-day mortality was comparable between the control and the intervention group at 10.9% and 10.8%, respectively. In our study, we reported proportions that received narrow-spectrum and broad-spectrum regimens, and the latter was reduced over the six-year study period from 34 to 17%, a relative reduction of nearly 50%. This result is in line with antimicrobial stewardship guideline recommendations, which favor interventions that target specific infectious syndromes such as CAP [13].

Adherence to CAP guideline recommendations is important. Several studies have provided evidence that adherence is efficacious and safe for the management of non-severe CAP [8, 12, 14, 15]. A 2017 meta-analysis showed that guideline-adherent prescriptions of hospital antimicrobial therapy increased from 43 to 58% after the implementation of stewardship interventions [12]. In our study, we noted that a wide range of empirical regimens were deployed in the ER setting at presentation, although a majority were narrow-spectrum antimicrobials. This could reflect diagnostic challenges in the ER setting, rather than non-adherence to guideline recommendations. The Dutch study also signals that even during study enrollment, physicians are reluctant to prescribe narrow-spectrum therapy for non-severe CAP, as evident by the low adherence in the control group [8]. It also showed that prescription habits during trial settings varied substantially between participating hospitals, as broad-spectrum antimicrobial therapy for non-severe CAP were unequally reduced, ranging from 17 to 39%. In our study, unwarranted and unjustified broad-spectrum antimicrobial therapy was frequently initiated and continued without a documented rationale. We were unable to point out specific reasons for this pattern. In microbiologically unconfirmed cases, we anticipate that clinical improvement while on a broad-spectrum regimen could explain reasons to defer transition. In addition, we also anticipate that if microbiologic tests provide definite or possible aetiology, several clinicians are still reluctant to make therapy transition as long as clinical improvement is observed. However, these hypotheses do not explain why non-severe CAP cases were prescribed broad-spectrum regimens in the first place.

Therapy for CAP was transiently deviating during the first pandemic year 2020, as a substantial larger proportion were prescribed cefotaxime. This could relate to insufficient evidence-based therapy recommendations for the treatment of COVID-19 with a possible concurrent bacterial superinfection at that time point. However, Norwegian health authorities rapidly deployed guidelines on COVID-19 management in April 2020. In the present study, we took active steps not to include lower respiratory tract infections with a definite or possible viral aetiology.

Adherence to CAP guideline recommendations also includes diagnostic strategies in order to provide definite or possible aetiology. We achieved a considerable increase from 33.7 to 56.2% in cases that were microbiologically confirmed. We have previously reported the results of an intervention study focusing on upscaling numbers and quality of lower respiratory tract secretions [9]. Our studies were conducted relying on standard laboratory protocols, and this leads us to conclude that interventional rather than new or extended laboratory services prevailed.

The assessment of disease severity has consistently played a key role to antimicrobial therapy approach in CAP [3]. This is also highlighted in Norwegian guideline recommendations. However, our study reveals that documented severity assessment is generally lacking and therefore under-communicated, maybe relying on a more pragmatic definition that CAP patients admitted to the ICU constitute severe cases.

De-escalation strategies are heavily recognized as a part of the global antimicrobial stewardship approach [16, 17]. Transition to narrow-spectrum, targeted antimicrobial therapy, and the timely conversion to oral formulations, were frequently unutilized among included cases in our study. Written documentation of such assessments was uniformly lacking. The optimal de-escalation strategies are to some extent studied in the ICU setting and in mechanical ventilated patients [18]. For non-severe CAP, as for several other non-severe infections, the impact of stewardship measures depends on many human and organizational factors [19, 20]. Despite evidence-based approach to target de-escalations strategies, barriers to guideline adherence exist among physicians. A more efficient strategy to overcome these barriers may be to tailor interventions to human behavioral change [20].

Therapy duration exceeded guideline recommendations substantially in our study. Comparable findings are previously reported [21–23]. The rationale for shorter duration (< 6 days) for CAP, regardless of disease severity, proved non-inferior to longer (> 7 days) in a recent meta-analysis comprising 21 trials which included over 4.000 patients [24]. A recent, double-blind, randomized, placebo-controlled, non-inferiority trial from France showed that 3 days of beta-lactam therapy plus 5 more days of placebo, was non-inferior to 5 more days of amoxicillin-clavulanate, for CAP episodes that met clinical stability criteria [25]. Cure rates at day 15 were 77% and 68%, respectively, and incidences for adverse events were comparable. The implementation of certain clinical stability criteria in CAP, has previously been shown in a large multicenter, non-inferiority, randomized clinical trial to be a safe strategy when assessing therapy duration [26]. The observed therapy duration in our study warrants new approaches to understand reasons for deferring antimicrobial cessation.

Our observational study has limitations and some strengths. The nature of the collected data did not allow for timing of antimicrobial therapy, only dates. In addition, the availability of clinical evaluations and reasoning on empirical and targeted therapy, microbiology results, susceptibility results, disease severity, and de-escalation strategies, were largely dependent on what attending physicians found necessary to document. This may contribute to missing or misinterpretation of data. The observational nature of the study does not unequivocally state the presence of a causal relationship between the intervention implemented and the CAP management results. Previous studies have documented that a substantial proportion of patients admitted for lower respiratory tract symptoms resembling CAP, and received antimicrobial therapy for this, ultimately are diagnosed with non-infectious causes [27]. To minimize this, and to avoid cohort heterogeneity, we applied several, strict exclusion criteria. In addition, in the Dutch study, only 25% of included CAP episodes were radiologically confirmed [8], as opposed to 91% in our study. We agree that several important questions remain unanswered in CAP management, especially microbiological confirmation strategies, the role of molecular technology, and therapy optimization [28], and that randomized clinical trials are needed to clarify optimal management. However, we support observational strategies to provide effectiveness studies on guidelines adherence at an everyday clinical practice level, outside of trial settings [29].

In conclusion, antimicrobial therapy for CAP at our teaching university hospital was frequently non-adherent to national clinical practice guideline recommendations. Non-adherence was particularly frequent for empirical regimens in non-severe CAP, for the timely de-escalation to targeted antimicrobial therapy, for the timely transition to oral regimens, and for overall therapy duration. However, adherence was to a large extent modifiable. The study reaffirms CAP as an important target for antimicrobial stewardship, and CAP seem suitable for interventions to increase adherence.

## Electronic supplementary material

Below is the link to the electronic supplementary material.

Supplementary Material 1

Supplementary Material 2

## Data Availability

The datasets generated and analysed during the current study are not publicly available due to large file sizes and complex registrations but are available from the corresponding author on reasonable request.

## Declarations

### Transparency declaration

None to declare.
