# Supplementary material for: Antimicrobial therapy of community-acquired pneumonia during stewardship efforts and a coronavirus pandemic: an observational study
Source: BMC Pulm Med. 2022 Oct 14;22:379. doi: 10.1186/s12890-022-02178-6 (PMC9569007; doi:10.1186/s12890-022-02178-6)
Supplement: Supplementary file 1 — Supplementary Material 1 [file 12890_2022_2178_MOESM1_ESM.docx]

Norwegian National Clinical Practice Guideline Recommendations 2013

# Community-acquired pneumonia

| Setting | Antimicrobial regimen | Comments | Duration |
| --- | --- | --- | --- |
| Empirical standard regime | Benzylpenicillin 1.2 g x 4 IV | Early oral conversion to phenoxymethylpenicillin 1 g x 4 PO or amoxicillin 500 mg x 3 PO | 5-7 days |
| Severe CAP  (as evident by CRB65 3-4 and respiratory failure) | Benzylpenicillin 1.2 g x 4 IV and gentamicin 5 mg/kg x 1 IV, or cefotaxime 1-2 g x 3 | Addition of macrolide if clinical suspicion of *Mycoplasma pneumoniae* or *Legionella* spp | 7-10 days |
| If beta-lactam allergy (type 1) | Erythromycin 500 mg x 4 IV or clindamycin 600-900 mg x 3-4 IV |  |  |
| If beta-lactam allergy (non-type 1) | Cefuroxime 1.5 g x 3 IV |  |  |

Norwegian National Clinical Practice Guideline Recommendations 2021

# Community-acquired pneumonia

| Setting | Antimicrobial regimen | Comments | Duration |
| --- | --- | --- | --- |
| Mild-moderate CAP | Benzylpenicillin 1.2 g x 4 IV | If beta-lactam allergy type 1: Erythromycin 500 mg x 4 IV | 5 days |
| Severe CAP  (as evident by CRB65 3-4 managed at ward level) | Benzylpenicillin 1.2 g x 4 IV and gentamicin 5 mg/kg x 1 IV | If beta-lactam allergy type 1: Clindamycin 600 mg x 3 and ciprofloxacin 400 mg x 3 IV  If beta-lactam allergy non-type 1, kidney failure or pregnancy: Cefotaxime 2 g x 3 IV | 7 days |
| Severe CAP  (as evident by CRB65 3-4 managed in the ICU) | Cefotaxime 2 g x 3 IV and ciprofloxacin 400 mg x 3 IV | Piperacillin-tazobactam 4/0.5 x 4 IV and ciprofloxacin 400 mg x 3 IV are considered equivalent | 7 days |
