# Supplementary material for: Antimicrobial therapy of community-acquired pneumonia during stewardship efforts and a coronavirus pandemic: an observational study
Source: BMC Pulm Med. 2022 Oct 14;22:379. doi: 10.1186/s12890-022-02178-6 (PMC9569007; doi:10.1186/s12890-022-02178-6)
Supplement: Supplementary file 2 — Supplementary Material 2 [file 12890_2022_2178_MOESM2_ESM.docx]

Antimicrobial stewardship interventions

# Antimicrobial stewardship team

St. Olavs hospital trust established an antimicrobial stewardship team in 2016. The team has the following staffing:

| Role | Speciality | Comment |
| --- | --- | --- |
| Leader | Infectious diseases specialist | 6 years as a leader |
| Member | Infectious diseases specialist, microbiologist | 6 years as member |
| Member | Microbiologist, infection control specialist | 6 years as member |
| Member | Pharmacology specialist | 6 years as member |
| Member | Microbiologist | 2 years as member |
| Member | Clinical pharmacist | 6 years as member |
| Member | Pharmacist | 6 years as member |
| Member | Pharmacist | 1 year as member |

# National clinical practice guideline recommendations

Guideline recommendations was available through website from national health authorities. Both 2013 version and 2021 version of all chapters adapted and transferred to internal hospital procedures, and implemented as standard of care.

## Implementation steps taken:

- 2016
  - Review lecture to all available emergency room staff
  - Handouts of pocket version of clinical practice guidelines
  - Posters of selected clinical practice guideline recommendations
- 2020
  - Review lecture to all available emergency room staff
  - Handouts of pocket version of clinical practice guidelines
  - Posters of selected clinical practice guideline recommendations

# Tutoring sessions

Antimicrobial stewardship team offered scheduled sessions for all inclusion periods through years from 2016-2021. The ER department and departments within medical and pulmonary fields were visited yearly inn selected periods through March-May.

## Activities

- On site tutoring sessions (prospective audit with feedback) for 2-3 hours daily in the ER setting
- Focus on
  - Disease severity
  - Microbiological sampling methods
  - Empirical antimicrobial therapy
- Patient care rounds (prospective audit with feedback) in selected medical and pulmonary wards for 1 hour daily
- Focus on
  - Microbiological testing reports
    - Acknowledging, practical use, interpretation
  - De-escalation strategies, including transfer to targeted antimicrobial therapy and oral formulations
  - Therapy duration
    - According to clinical practice guideline recommendations

# Performance statistics

The antimicrobial steward team provided a yearly report on performance to involved departments at a ward-level.

## Reported statistics

- Antimicrobial consumption for selected antimicrobial agents
- Number of microbiological samples used in CAP management
  - Upper respiratory tract samples
  - Lower respiratory tract samples
  - Other samples
- Diagnostic yield of samples
- Antimicrobial resistance prevalence
- Academic detailing
  - Performance on the last 20 patients managed in selected departments
    - Correctly performed microbiological sampling
    - Empirical antimicrobial therapy in accordance with clinical practice guideline recommendations
